# Viral lysis and host reprogramming impact carbohydrate, amino acid, and osmolyte cycling in salt-marsh tidal creek sediments

**DOI:** 10.1093/ismeco/ycag184

**Published:** 2026-07-01

**Authors:** Riccardo Frizzo, Silvia Pettenuzzo, Enrico Bortoletto, Irene Gregori, Alessandro Vezzi, Mattia Panin, Sciamila Hemmati, Lorenzo Archetti, Stefano Mammi, Sara Bogialli, Paola Venier

**Affiliations:** Department of Biology, University of Padova, Via Ugo Bassi 58/B, 35131 Padova, Italy; Department of Chemical Sciences, University of Padova, Via Marzolo 1, 35131 Padova, Italy; Department of Biology, University of Padova, Via Ugo Bassi 58/B, 35131 Padova, Italy; Department of Biology, University of Padova, Via Ugo Bassi 58/B, 35131 Padova, Italy; Department of Biology, University of Padova, Via Ugo Bassi 58/B, 35131 Padova, Italy; Department of Biology, University of Padova, Via Ugo Bassi 58/B, 35131 Padova, Italy; Department of Biology, University of Padova, Via Ugo Bassi 58/B, 35131 Padova, Italy; Department of Chemical Sciences, University of Padova, Via Marzolo 1, 35131 Padova, Italy; Department of Chemical Sciences, University of Padova, Via Marzolo 1, 35131 Padova, Italy; Department of Chemical Sciences, University of Padova, Via Marzolo 1, 35131 Padova, Italy; Department of Biology, University of Padova, Via Ugo Bassi 58/B, 35131 Padova, Italy

**Keywords:** salt marsh, sediment, metagenomics, metabolomics, viromics, viral lysis, host reprogramming, genome-scale models, auxiliary viral genes

## Abstract

Salt marshes are highly productive ecosystems where microbial communities drive key transformations of organic matter at rates often exceeding those of oceanic and inland environments. Viruses are recognized as important drivers and regulators of global biogeochemical cycling, yet their diversity, host range, and functional roles in salt marsh ecosystems remain largely unresolved. To address these gaps, we investigated how viral lysis and host reprogramming can affect microbe-mediated organic matter transformations in a salt marsh of the Venice lagoon (Italy). Focusing on tidal creek surface sediments, we reconstructed 311 metagenome-assembled genomes (MAGs), built corresponding genome-scale metabolic models (GEMs) individually constrained with 121 metabolites detected in the sediments, and identified 3537 viral populations (vOTUs) across 10 samples. To assess the impact of viral lysis, we inferred prokaryotic hosts for 243 vOTUs and analysed host metabolism through MAG pathway analysis and GEM flux modelling across 13 bacterial orders, thus highlighting a negative impact on polysaccharide degradation, organic nitrogen mineralization, and organosulphur mineralization/volatilization processes. For host metabolic reprogramming, we characterized a subset of 50 auxiliary viral genes (AVGs) by mapping them to GEM reactions and analysing their stoichiometry, directionality, and pathway context, outlining two dominant strategies: resource scavenging through nucleotide–sugar biosynthesis, amino acid utilization, and sulphate assimilation; functional host maintenance through cofactor biosynthesis, electron transport, and energy production through carbonyl-compound utilization. Our findings provide a mechanistic view of the viral influence on organic matter transformations in salt marsh sediments and confirm viruses as key players in salt marsh biogeochemistry.

## Graphical Abstract

**Graphical Abstract:**
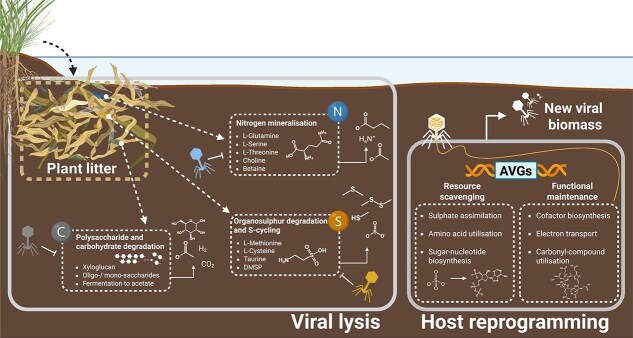

## Introduction

Salt marshes are among the most active and carbon-rich ecosystems on Earth, where halophilic plants and dynamic microbial communities are shaped by nutrient inputs and by diel, tidal, and seasonal rhythms. These conditions drive element cycling rates that often exceed those of many oceanic and inland environments [1–7]. However, salt marshes are under global threat, with an estimated annual area loss of 0.28%, due to sea-level rise, subsidence, wave erosion, and anthropogenic disturbance [3, 7, 8].

Understanding soil organic matter (SOM) transformations in salt marshes is crucial to fully understanding carbon cycling in coastal wetlands and better estimate blue-carbon budgets [9] and ecosystem services [10]. In salt marshes, SOM and inorganic compounds contribute directly to vertical accretion, particularly in the upper layers (0–20 cm), thereby supporting carbon storage [11]. In this context, microbial activity drives the maintenance of nutrient pools [12], SOM formation, and the generation of recalcitrant residues then stabilized on mineral surfaces or within aggregates [7, 13, 14]. Current evidence indicates the capacity of salt marsh microbes to grow on a dynamic mixture of fermentation products (e.g. short-chain carboxylic acids, alcohols) [15], methylated osmolytes (choline, betaine) [16, 17], carbohydrates, amino acids, and complex polymers (hemicellulose, lignin) [18, 19]. However, genome-resolved datasets are limited for salt marsh sediments, and it remains unclear how these substrates are utilized and transformed at the community level [20, 21].

While prokaryotes and microeukaryotes participate in SOM transformations, viruses are increasingly recognized as important regulators of SOM availability and utilization. Microcosm experiments showed that active viral lysis can enhance microbial metabolism through the reuse of cell lysate (viral shunt) and can increase the abundance and humification of recalcitrant organic matter, promoting long-term SOM retention (viral shuttle) [22]. Moreover, during host infection, viruses can redirect cell processes by altering the expression of regulatory proteins or promoting host physiology through auxiliary viral genes (AVGs) [23]. Studies in peatlands with permafrost thaw and mangrove soils highlighted how virus-encoded auxiliary metabolic genes (AMGs, a type of AVG) can modulate the transformation of plant-derived polysaccharides into labile monosaccharides and short oligosaccharides [24, 25], the utilization of amino- and carboxylic-acid, and sulphur metabolism [26, 27]. Although information on virus diversity and distribution in salt marshes is gradually expanding [28–30], it is unclear which microbial lineages are most affected by viral lysis, and how viral reprogramming can alter host functions.

**Figure 1:**
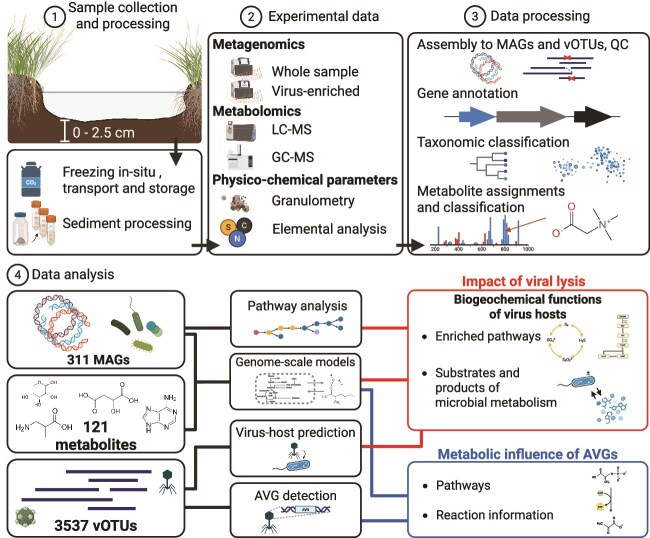
Study workflow. From the upper-left to the bottom-right corner, the figure provides an overview of sample collection, experimental data acquisition, data processing, and integrative analysis steps. Abbreviations: LC-MS/GC-MS, liquid-chromatography/gas-chromatography mass spectrometry; MAG, metagenome-assembled genome; vOTU, viral operational taxonomical unit; QC, quality control; AVG, auxiliary viral gene.

To investigate the role of viruses on microbe-mediated SOM transformations, we sampled tidal creek sediments (0–2.5 cm depth) of a reference salt marsh in the Venice Lagoon, analysing sediment-associated metabolites, prokaryotes, and viruses. Specifically, we used metagenomics, metabolomics, and viromics data to build genome-scale metabolic models and identify virus–host pairs, as a basis to estimate the impact of viral lysis on microbial SOM transformations and AVG-mediated reprogramming on host metabolism (Fig. 1).

## Materials and methods

All statistical analyses, data manipulation, and visualization steps were performed in R 4.4.2 [31]. Multiplot figures were arranged using Biorender (www.biorender.com).

### Sample collection and processing

The contents of this section are reported in full detail in Supplementary file S1.

**Figure 2:**
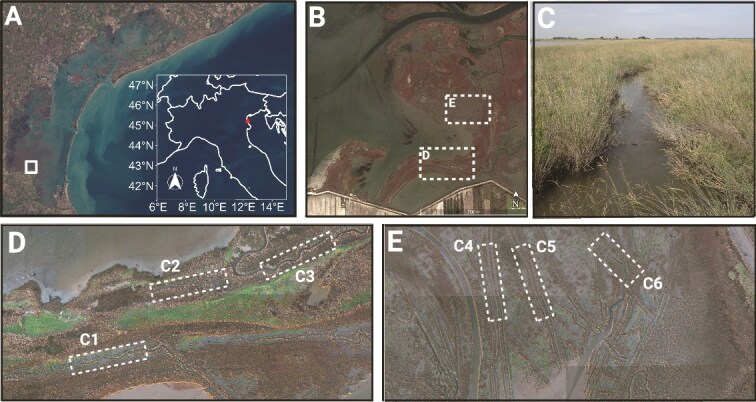
Geographical context of sampling sites. (A) Position of the Boschettona salt marsh in the Venice lagoon. (B) Satellite image of the area in 2021 (source: Google earth). Sampling areas D and E are delimited by dotted white lines. (C) Example picture of a tidal creek. (D, E) Composite drone images of the sampled tidal creeks, with sampling sites delimited by dotted white lines. In detail, we characterized sediment samples from six tidal creeks. All sites were surrounded by salt marsh vegetation, mostly *Sporolobus* spp., *Limonium* spp., and *Sarcocornia/Salicornia* spp. At the time of sampling, the local tide level ranged from 0 to 0.30 m (diel tide fluctuations nearby the Boschettona area were reported to be 0.5–0.6 m at salt marsh banks and 0.2–0.4 m in the inner creeks [32]).

Sediment samples (0–2.5 cm depth) were collected in June 2023 from tidal creeks of the Boschettona salt marsh (Venice lagoon, IT) across six sampling points in duplicate (Fig. 2). For CHNS elemental analysis, sediments were dried overnight at 100°C, ground, and ~15 mg were transferred into tin capsules and added with vanadium pentoxide; TOC fractions were additionally treated with 10% HCl to remove inorganic carbon. Analysis was performed on a Flash Smart instrument (Thermo Scientific). For granulometry, ~30 g of wet sediment was treated with 15% hydrogen peroxide, added with sodium hexametaphosphate, dried, and ground prior to analysis on an LS 13320 XR laser granulometer (Beckman Coulter), with data classified into Wentworth grain size classes. For ICP-MS, ~100 mg of freeze-dried sediment was digested in HNO_3_ at 100°C, diluted to 5% HNO₃ with ^72^Ge as internal standard, and filtered on PTFE membranes before analysis on an Agilent 7700 ICP-MS for 30 elements. For LC-MS, 1 g of sediment was extracted with methanol:water (3:1) and chloroform, and the aqueous fraction filtered on 0.2 μm regenerated cellulose membranes before injection onto an Ultimate 3000 UHPLC coupled to a Q-Exactive Orbitrap (Thermo Fisher Scientific) in HILIC mode, acquiring data in positive and negative ESI modes.

For GC-MS, 2 g of homogenized sediment was subjected to HiSorb sorptive extraction (DVB/CWR/PDMS fibre, 60 min at 60°C) on a Centri platform (Markes International), with volatiles analysed on an Agilent 8860 GC coupled to an Agilent 5977B MSD.

Raw data from LC/GC-MS were processed with MS-DIAL 4.9, metabolites were identified at level 2 against authenticated spectral libraries, and statistical analyses were performed in MetaboAnalyst 6.0.

For metagenomics, DNA was extracted from ~500 mg of wet sediment using the DNeasy PowerSoil kit, sequenced by paired-end 150 bp DNBSEQ, quality-filtered with SOAPnuke, and co-assembled with Megahit within the SqueezeMeta pipeline.

For viromics, virus-like particles were enriched from 250 g of wet sediment per sample by sequential centrifugation, 0.45 μm filtration, and PEG precipitation, followed by DNase treatment and DNA extraction with the ZymoBIOMICS kit. Three samples yielded sufficient library quality for paired-end 100 bp sequencing; reads were quality-filtered with SOAPnuke and individually assembled with Megahit.

### Metagenomic analysis, metabolic modelling, and integration with metabolomics

Metagenomic reads from each sample were mapped against contigs using coverM 0.7.0 (*contig* module) [33]. Contigs and associated mapping data were then used for binning with SemiBin2 2.1.0 (*Easy co-assembly* mode) [34]. Bin quality was assessed with CheckM2 1.0.1 (*predict* module) [35], bins with completeness ≥70% and contamination ≤10% were retained for downstream analysis as metagenome-assembled genomes (MAGs) [36]. MAGs were dereplicated at 99% ANI using Galah 0.4.0 (*cluster* module *--ani 99 --checkm2-quality-report*) [37] to retain strain-level accessory gene content useful for virus-host predictions [38]. The taxonomic classification of MAGs was performed with GTDB-Tk v2.4.0 (*classify_wf,* database revision 220) [39]. Metagenomic reads were mapped against MAGs to obtain Reads-per-Million (RPM) counts, using coverM (*genome* module) [33]. RPM values were normalized via Hellinger transformation and reported as relative abundance (%). MAGs were added to our local copy of the iPhoP phage host database (*add_to_db* function, database version *iPHoP_db_Aug23_rw*) to generate a custom host database [38].

Genome-scale metabolic models (GEMs) were built from each MAG using gapseq 1.2 [40]. Reactions and transporters were predicted with the functions *gapseq find* and gapseq *find-transport* and used as input for *gapseq draft* to construct the draft models; all steps were run with default parameters. The final step of model construction in gapseq (gap-filling) adds reactions to the model that enable biomass production and recover likely metabolic capabilities when sequence similarity to known reference genes is near but does not reach the similarity threshold. Gap-filling of a draft model in gapseq requires a growth medium ideally known to support the organism’s growth *in vivo* [40]. We predicted the medium composition of each draft GEM with *gapseq medium*, using as input the metabolites identified via LC-MS and GC-MS, and assigning to each metabolite a *maxFlux* value proportional to its peak area, scaled from 0.1 to 10 mmol g_DW_^−1^ h^−1^ (Supplementary File S2). The *gapseq medium* function estimated the optimal medium composition and the metabolite uptake limits for each GEM, based on the reaction network and the identified metabolites. This approach allowed the integration of sediment characteristics into the models, thus reducing the uncertainty of predictions [41, 42]. Draft GEMs were then gap-filled with *gapseq fill* using the corresponding metabolome-informed media.

### Pathway enrichment and metabolite utilization analysis

Enrichment of metabolic pathways by taxonomic order was assessed from the output of *gapseq find*, using a two-sided Fisher’s exact test. Only bacterial orders represented by more than five MAGs were tested. For each pathway, *P*-values were corrected for multiple comparisons across orders using Benjamini–Hochberg false discovery rate (BH). Pathways were considered significantly enriched in a bacterial order when BH-adjusted *P* < .05 and odds ratio > 2. Among enriched pathways, those linked to substrate utilization were further analysed.

For metabolite utilization, Flux Balance Analysis (FBA) was performed for each GEM using cobraR v0.1.1 [43, 44]**,** solving for biomass growth (biosynthesis of molecular constituents in a right proportion for cell replication [40]). Bacterial orders with at least 5 GEMs and metabolites exchanged in at least 10 GEMs were considered in downstream analyses. We summarized metabolite exchanges by taxonomic order, calculating exchange frequency (proportion of GEMs importing or exporting each metabolite) and exchange flux (median import/export flux, negative = import, positive = export). Metabolite import or export fluxes aggregated by order were tested against the median value of the whole set (Wilcoxon’s Rank sum test, one-sided, *P* < .05 ‘FDR-adjusted’).

### Identification of viral sequences, taxonomy assignment, host prediction, and gene function analysis

Viromics and metagenomics contigs were pooled into a single file (contig pool). Virsorter2 2.2.4 (*include-groups: ‘dsDNAphage, ssDNA’*) [45] and geNomad 1.7.3 (*end-to-end* workflow*,* filtering preset: *conservative*) [46] were used to screen viral sequences in the contig pool. The quality of the viral sequences identified was assessed using CheckV v1.0.3 (*end_to_end* workflow) [47]. Summary tables from Virsorter2, geNomad, and CheckV were merged and used for selecting viral sequences as follows: (i) contigs longer than 5 kbp, having at least one viral gene, less than 2 host genes, and a geNomad virus score > 0.8 or Virsorter2 score > 0.8 or CheckV completeness >70; (ii) contigs shorter than 5 kbp and having CheckV completeness >70. Passed sequences were classified as viral or proviral based on geNomad and CheckV classification and split into separate files. Viral and proviral sequences were independently dereplicated at 95% ANI over 85% of the aligned fraction, relative to the shorter sequence, using dRep 3.4.2 (*dereplicate* module, -pa 0.9 -sa 0.95 -nc 0.85 -cm ‘larger’) [48, 49]. Dereplicated viral and proviral contigs were considered as independent viral operational taxonomic units (vOTUs). Metagenomics reads were mapped against vOTUs to obtain Reads-per-Million counts (RPM), using coverM (*contig* module) [33] and adopting 3 RPM as the minimum threshold value. RPM values were normalized via Hellinger transformation, and reported as relative abundance (%). The taxonomic classification of vOTUs was performed with geNomad and vContact 3 3.0.0b61 [50], prioritizing vContact’s taxonomy in case of conflicting results. vOTUs were assigned to gene-sharing clusters applying the function *components* (igraph package [51]) on vContact3 graph output; the gene-sharing network was visualized with r-igraph [51]. Host taxonomy and vOTU-MAG associations were determined with iPhoP (*predict* model) [38] using the custom phage–host database. vOTUs genes were annotated using pharokka 1.7.3 (--meta) [52] and phold 0.2.0 [53] sequentially. vOTUs genes belonging to categories ‘Auxiliary metabolic genes and host takeover’ and ‘Other’ were selected as putative auxiliary viral genes (AVGs). These AVGs were manually checked, excluding functions linked to virus replication and host interaction (e.g. transport, secretion systems, translation repressors, resistance proteins, DNA/RNA metabolism, and glycan modification), and genes at the immediate edge of a viral scaffold, which may represent possible host contaminations. The final set of AVGs was searched in MAGs by amino acid sequence similarity with BLAST+ 2.14.1 (blastp function) [54]. Hits with a percentage of identity ≥30% over at least 70% of the query and e-value <10^−5^ were retained. The genomic coordinates of the resulting BLASTp hits were intersected with the CDS positions of each MAG reported by gapseq using *bedtools intersect* v2.31.1 [55], thus linking the matching AVGs to the corresponding metabolic reaction(s) in GEMs.

## Results and discussion

### Sampling sites and abiotic composition of sediments

**Figure 3:**
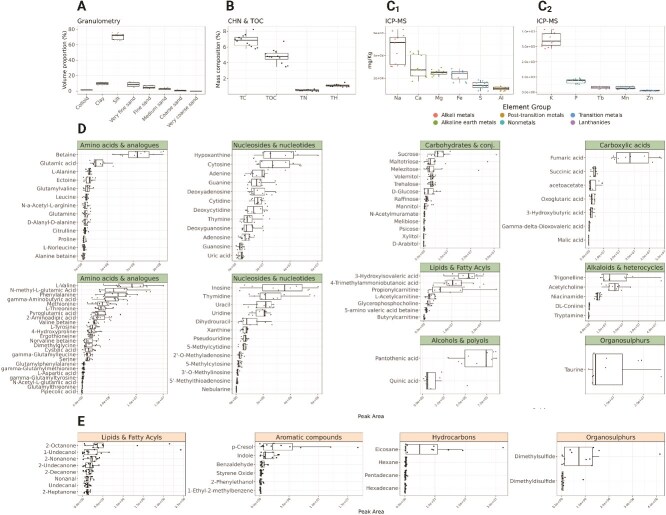
Composition of salt-marsh tidal creek bulk sediments. (A) Granulometric fractions. (B) Total and organic carbon, total nitrogen, and hydrogen content. C_1,2_ concentrations in mg/kg of the most abundant elements. (D, E) Metabolite profiles from LC- and GC-MS, reported as absolute peak area (procedural details in Materials and Methods and Supplementary File S3—S1–S4).

Sediments in salt-marsh tidal creeks contribute significantly to wetland carbon storage and are focal points for heterotrophic and chemolithoautothrophic metabolism [20, 56]. The surface sediments of tidal creeks (Fig. 2) were mostly composed of silt, clay and very fine sand (71%, 10%, 9%, respectively); the CHNS ratio was 13:2:1:1, and 70% of the total carbon was organic (Fig. 3A and B, Supplementary File S3—S1). Elemental analysis revealed elements at concentrations exceeding 30 mg/kg in all sediment samples: alkali and alkaline earth metals (Na, Ca, Mg, Sr, K), transition metals (Fe, Mn, Zn), post-transition metals (Pb), non-metals (P, S), and the lanthanide Tb (Fig. 3C_1_, 3C_2_, Supplementary File S3—S2). The organic C fraction and the prevalence of elements like Na, Ca, Mg, Fe, S, K, P, Mn, Sr and Si are consistent with low marsh sediments influenced by surrounding vegetation and daily tidal inundation [57]. Indeed, 121 organic molecules were analytically detected in the same sediments (Fig. 3D and E, Supplementary File S3—S3, S4): 101 water-soluble compounds by LC-MS (15% of 6769 total features) and 20 volatile compounds by GC-MS (36% of 212 total features). The most frequent LC-MS signals identified amino acids, carbohydrates, purines, and pyrimidines. Amino acids prevailed in terms of peak area (PA), followed by purine and pyrimidines, fatty acids, and carbohydrates. The metabolites with the highest average PA were betaine, L-glutamic acid, hypoxanthine, L-alanine, ectoine, cytosine, L-glutamylvaline, L-leucine, N-α-acetyl-L-arginine, adenine, trigonelline, and several γ-glutamyl-amino acids. The most frequent GC-MS signals identified carbonyl-containing aliphatic compounds, followed by alkanes and benzenes. Eicosane, p-cresol, indole, and dimethyl sulphide had the highest average PA.

### Microbial and viral diversity

**Figure 4:**
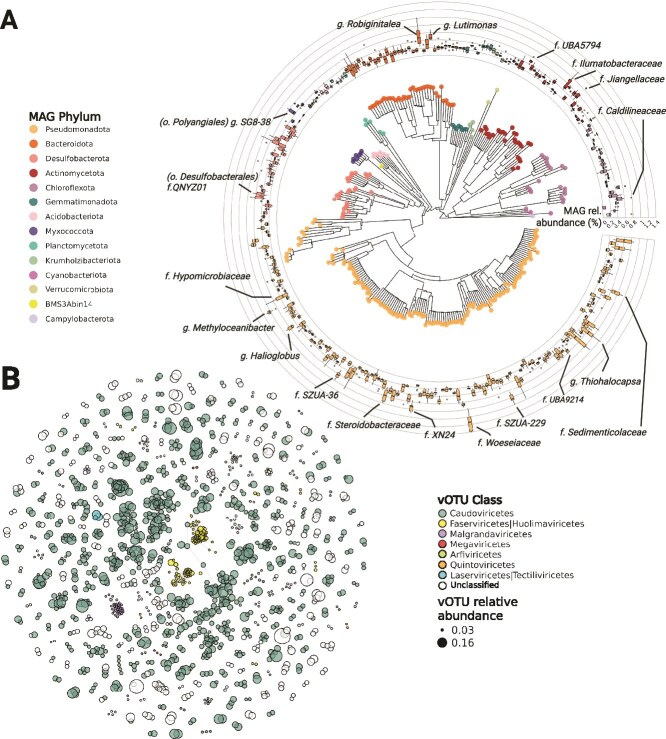
Microbial and viral taxonomy with corresponding relative abundances in salt-marsh tidal creek sediments. (A) Pruned GTDB reference tree (bac120) showing 310 bacterial metagenome-assembled genomes (MAGs); one archaeal lineage is not shown. Tree tips indicate phylum assignments. Outer boxplots show the distribution and relative abundance of MAGs across the 10 samples, and labels around the boxplots highlight family- or genus-level assignments for the most abundant MAGs. (B) Gene-sharing network of all non-singleton vOTUs. Nodes represent vOTUs and are connected by edges when they share genes, resulting in clustered groups. Nodes are coloured by class-level taxonomy, node size is proportional to the average relative abundance whereas the distance between two nodes decreases as the shared genes increase. The number of node clusters having the same colour highlights the diversity of vOTUs within a taxonomical class.

Metagenomic sequencing produced an average of 114.2 million reads per sample (read length 150 bp; Phred score > 24, Supplementary File S4—S3). Read assembly yielded 5.26 million contigs whose taxonomical annotation revealed 70 archaeal, 467 bacterial, and 190 eukaryotic lineages (Supplementary File S5). Contig binning, quality filtering, and dereplication led to the construction of 311 MAGs with mean completeness 85.2 ± 8.5% and contamination 3.9 ± 2.3%; genome size of 3.47 ± 0.93 Mbp (1.3–6.8 Mbp); GC content of 58.6% (36%–73%); and median number of CDS 3338 ± 734. Overall, 19.3 ± 2.8% of the metagenomics reads mapped to the 311 MAGs. Taxonomy assignments revealed 310 bacterial genomes and 1 archaeal genome, together encompassing 15 phyla: predominantly *Pseudomonadota* (155), *Bacteroidota* (35)*, Desulfobacterota* (32), *Actinomycetota* (28), and *Chloroflexota* (23) (Fig. 4A, Supplementary file S6—S1). Weighted by relative abundance, the 311 MAGs were dominated by heterotrophs, primarily within *Pseudomonadales (*8.5%*,* mainly *Haliaceae), Woeseiales (6.3%),* and *Bacteroidales (4.3%)*. Sulphur-utilizing bacteria were also well represented by *Desulfobacterales (6.3%)*, *Desulfobulbales* (3.3%), and *Acidimicrobiales (2.7%)*. *Chromatiales* included recognized mixotrophs (14.1%, e.g. *Sedimenticolaceae*, *Chromatiaceae*). Some of the most abundant individual MAGs belonged to methylotrophic lineages, such as *Methyloligellaceae* (*Methyloceanibacter*) and *Methylomonadaceae* (Fig. 4A). Such a community composition is consistent with a recent metagenomic survey reporting *Pseudomonadales*, *Desulfobacterales*, *Desulfobulbales*, *SZUA-365* and other *Bacteroidales* members, and *UBA6911* acidobacteria [58] in the surface sediments of natural salt marsh sites [30]. These and other prokaryotic taxa, including *Chromatiales*, were previously detected in salt marsh sediments by 16S rRNA gene barcoding [59]. Forty-two families of undefined lignocellulolytic bacteria were identified in a UK salt marsh community dominated by *Gammaproteobacteria*, *Bacteroidetes,* and *Deltaproteobacteria* [19], whereas *Woeseiales* of coastal surface sediments were described as chemoorganoheterotrophs and facultative chemolithoautotrophs consuming complex detrital matter, owing to the oxidation of hydrogen and inorganic sulphur compounds [30, 60].

As regards viruses, we obtained three virus-enriched samples, with an average of 175.9 million reads each (read length 100 bp; Phred score > 24). Merging the resulting assemblies to the metagenomic contigs yielded a combined dataset of 3806 vOTUs with an average size of 9.48 ± 7.68 kbp (54% of all vOTUs assembled from metagenomes, and 46% from viromes). On average, 0.39 ± 0.01% of the metagenomic reads and 5.1 ± 4.8% of the viromics reads did map to the vOTUs. We retained 3537 vOTUs mapping at least 3 RPM in metagenomics reads. A large fraction was unknown (42%), whereas the assigned vOTUs belonged to the realms of *Duplodnaviria*, *Monodnaviria,* and *Varidnaviria*: *Uroviricota* (51%, class *Caudoviricetes*) and *Phixviricota* (5%, *Malgrandaviricetes*) were the main phyla, whereas *Cressdnaviricota* (0.7%, *Repensiviricetes*), *Cossaviricota* (0.2%, *Quintoviricetes*), and *Nucleocytoviricota* (0.2%, *Megaviricetes*) were less represented (Fig. 4B, Supplementary file S6—S2).

We grouped 1461 vOTUs into 277 gene-sharing clusters using vContact3: the largest clusters included 103 and 88 vOTUs assigned to *Caudoviricetes* alongside 69 and 48 vOTUs identified as *Faserviricetes|Huo-limaviricetes|Malgrandaviricetes,* whereas small clusters (2–10 vOTUs) and singletons accounted for 953 and 2076 vOTUs, respectively (Fig. 4B). CheckV identified 2.7% of vOTUs as putative prophages. Wetlands are comparatively richer in viruses than other soils [61, 62], and the dominance of *Caudoviricetes* with evidence of novel, unclassified, vOTUs is a common finding. Following a metagenomic approach, nearly 82% of the 769 vOTUs identified in the rhizosphere of the salt-marsh plant *Spartina alterniflora* were classified within *Caudoviricetes*, representing novel genera not previously described [28], and the majority of the 587 vOTUs identified in Galveston Bay marshes (TX, USA) were assigned to unclassified orders of *Caudoviricetes* or remained unknown [30].

### Host predictions for vOTUs

**Figure 5:**
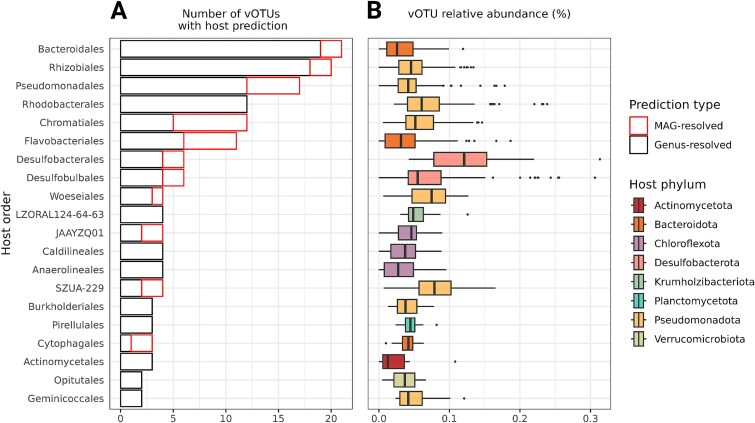
Number and relative abundance of vOTUs listed by host order. (A) Number of vOTUs predicted per host order (orders with at least two vOTUs are shown); order- and MAG-level predictions are shown. (B) Relative abundance of vOTUs across the 10 sediment samples by host order, coloured by host phylum.

To assess the influence of viral host infection and lysis on SOM, we identified the hosts targeted by the vOTUs using the iPhoP tool. We predicted hosts for 243 vOTUs. Among these, 123 vOTUs matched the taxonomy of our MAGs at the genus level and 41 vOTUs could be directly linked to 45 MAGs as virus–host pairs (Fig. 5A). The remaining 79 predictions involved taxa that could not be assembled into MAGs (mainly *Mycobacteriales* and *Lactobacillales)* indicating that host inference was not limited to assembled lineages*. Bacteroidales* and *Rhizobiales had* the highest number of associated vOTUs, followed by *Pseudomonadales, Chromatiales*, *Rhodobacterales*, and *Flavobacteriales* (Fig. 5A), whereas vOTUs of *Desulfobacterales, SZUA-229, Woeseiales,* and *Rhodobacterales* had the highest relative abundance (Fig. 5B). The number of vOTUs linked to each bacterial order showed a moderate positive correlation with the number of MAGs per order (Spearman’s rank correlation, ρ = 0.53, *P* < .001). This suggests either an overrepresentation of our MAG orders in the iPHoP database or a genuine Kill-the-Winner dynamics. The high relative abundance of vOTUs found associated with *Desulfobacterota* and *Pseudomonadota* reflects dominant hosts previously reported in bulk salt-marsh surface sediments [28, 30].

### Influence of viral lysis on salt marsh biogeochemistry

**Figure 6:**
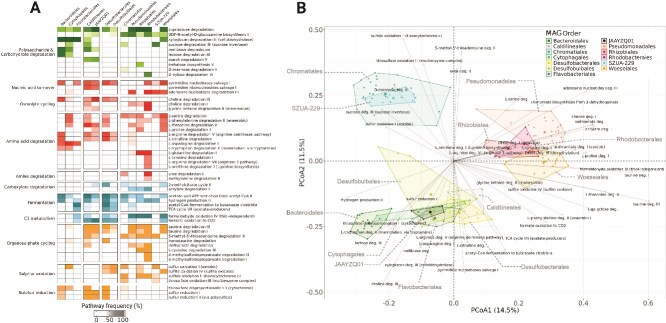
Pathways of substrate utilization enriched in the bacterial orders associated with vOTUs. (A) Heatmap displaying the distribution and frequency of enriched pathways. Filled cells indicate pathways detected in at least 25% of MAGs within an order. Top annotations report the order-level taxonomy of MAGs. Pathway categories and pathway annotations are shown on left and right, respectively. (B) Principal Coordinates Analysis (PCoA) of metabolic pathway presence/absence across genome-scale metabolic models (MAGs), based on Jaccard dissimilarity. Each point represents an individual MAG. Convex hulls delineate the ordination space occupied by each order. Radial arrows highlight a selection of substrate utilization pathways having squared correlation *r*^2^ ≥ 0.4 with the first two PCoA axes. Abbreviations: DMSP, dimethylsulfoniopropanoate; deg., degradation.

**Figure 7:**
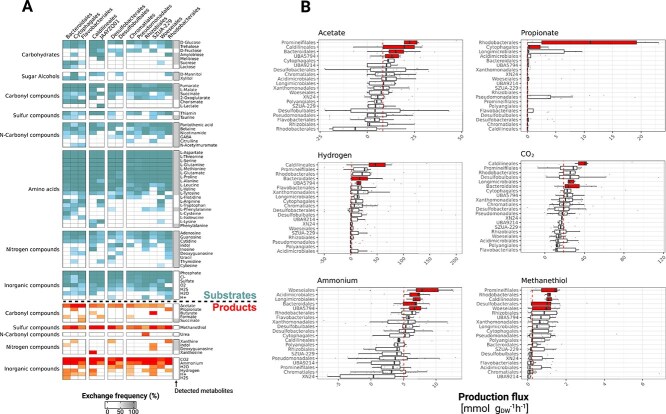
Predicted metabolite exchanges in bacterial orders associated with vOTUs. (A) Heatmap displaying the distribution and frequency of exchanged metabolites. Filled cells indicate metabolites predicted as substrates or products in at least 25% of GEMs within an order. Top annotations report the order-level taxonomy of GEMs. Classes and names of metabolites are shown on the left and right, respectively (those experimentally detected by LC-MS or GC-MS are marked in grey). (B) Exchange fluxes of main metabolic end-products across the top 20 orders in the community. The vertical red line indicates the community-wide median flux value. Filled boxplots indicate orders with production fluxes significantly higher than the community median (Wilcoxon rank-sum test, one-sided, FDR-adjusted *P* < .05).

Viruses can dramatically influence both microbial abundance and community structure through cell lysis, which is estimated to account for ~46% of prokaryote mortality in marine environments [63]. Viral lysis has strong implications for organic matter turnover at the ecosystem scale, and its biogeochemical significance arguably depends on the metabolic activities of the lysed hosts. In this study, we sought to pinpoint the microbial transformations influenced by viral infections in salt marsh sediments by examining the metabolic potential of the bacterial orders most frequently linked to vOTUs (Fig. 5). To this end, we first characterized the biochemical pathways of those orders (Fig. 6), focussing on the utilization and cycling of carbon, nitrogen, and sulfur compounds [64]. We then used the genome-scale models (GEMs), constructed from MAGs and gap-filled with metabolites detected in the sediment, to identify the substrates and products of bacterial metabolism, predicting which metabolite fluxes are most likely influenced by viral lysis (Fig. 7).

### Viral targeting of polysaccharide degraders influences carbohydrate availability

Members of *Bacteroidales, Flavobacteriales,* and *Cytophagales* were enriched in carbohydrate degradation pathways responsible for the saccharification of complex carbohydrates, reported for *Bacteroidota* [65, 66]. These included xyloglucan degradation (cellobiohydrolase), and lactose, melibiose, and trehalose degradation (Fig. 6A and B). Such degradative capacities are consistent with the net uptake of sucrose, lactose, trehalose, and sugar alcohols predicted for *Bacteroidota* GEMs (Fig. 7A). The detection of monomeric sugars (D-glucose, D-psicose, trehalose, D-mannose, N-acetylmuramate), oligomeric sugars (amylotriose, melibiose, melezitose, melitose, sucrose), and sugar alcohols (xylitol, arabitol, mannitol, volemitol) in the analysed sediments (Fig. 3) supports active microbial degradation of lignocellulosic matter: specifically, the polysaccharide component that accounts for the high rate of carbon mineralization in salt marsh sediments [67, 68]. The metabolite exchanges predicted through flux balance analysis position *Bacteroidales* GEMs among the most active fermenters, with fluxes of acetate, hydrogen, and carbon dioxide significantly above the community median (Fig. 7B). These terminal fermentation products also represent important substrates for chemolithoautotrophs such as *Desulfocapsaceae*, typical of deeper anoxic sediments [69].

*Bacteroidales* showed the highest number of associated vOTUs (Fig. 5A): although biases linked to database composition cannot be excluded [38], vOTU diversity within the same host order suggests virus diversification through arms-race dynamics [70]. Controlling bacteria competent for saccharification of complex carbohydrates, viruses modulate (likely decrease) the overall availability of labile saccharides and fermentation products to the rest of the community, affecting the rates of carbon mineralization and favouring the burial of plant organic matter into anoxic sediments [71, 72]*.*

### Infecting ammonia producers, viruses can limit ammonification rates

Salt marshes are often limited by nitrogen (N) availability and microbial N mineralization is fundamental for recycling reactive N back to primary producers [20]. In these ecosystems, N mineralization not only supports 50%–200% of plant demand [73] but also leads to N losses by tidal exchange [74]. The mineralization of organic nitrogen was a dominant process in the tidal creeks communities, with amino acids, osmolytes, and nucleotides serving as the primary nitrogen sources.

Organic nitrogen utilization differed across the bacterial orders linked to vOTUs: MAGs belonging to *Desulfobacterales*, *Desulfobulbales*, and *Bacteroidales* were enriched in *L*-arginine and *L*-citrulline degradation; *Rhizobiales* and *Rhodobacterales* in *L*-glutamine deamination; *Woeseiales*, *Rhizobiales,* and *Pseudomonadales* in *L*-threonine, *L*-serine, and *L*-proline degradation. Osmolyte catabolism represented a further ammonium source, with choline degradation enriched in *Bacteroidales* and *Rhodobacterales* and taurine degradation enriched in *Woeseiales*, *Pseudomonadales,* and *Desulfobacterales*. Additionally, *Rhodobacterales* showed capacity to utilize intermediates of amino acid catabolism, with the pathways of urea and methylamine degradation (Fig. 6A and B).

Metabolite exchanges predicted through flux balance analysis identified ammonium as the metabolite most broadly released in the community, with positive median fluxes from +3 to +8 mmol g_DW_^−1^ h^−1^ across the majority of MAG orders. Among vOTU-associated hosts, *Woeseiales* and *Bacteroidales* displayed levels of ammonium production above the community median (Fig. 7B), therefore acting as key contributors to nitrogen mineralization. In the metabolite profiles, nitrogen-containing compounds were the most represented chemical category (Fig. 3D), consistent with the substrates predicted by pathway and GEM flux balance analyses (Fig. 7A). Moreover, the detection of intermediate metabolites, including 5-oxoproline, acetylcholine, dimethylglycine, *L*-pipecolate, and tryptamine, supports active deamination and turnover of organic nitrogen (Fig. 3D).

In sum, two key groups of ammonifiers, *Woeseiales* and *Bacteroidales* present in tidal-creek sediments are likely under the influence of abundant and diverse viruses: viral modulation of these taxa may reduce rates of nitrogen mineralization, limiting nitrogen losses during excessive ammonification and therefore favouring retention and recycling organic nitrogen.

### Viral impact on organic sulphur mineralization and volatilization

Organosulphur compounds represent the second largest reservoir of sulphur in marine sediments, after pyrite [75, 76]. Here, two main organosulphur transformation processes were likely influenced by viral lysis: mineralization and volatilization.

For ‘organosulphur mineralization’, seven orders spanning *Pseudomonadota*, *Desulfobacterota*, and *Chloroflexota* were enriched in taurine degradation pathways (Fig. 6A and B), including *Desulfobacterales*, *Woeseiales,* and *Rhizobiales*, all linked to abundant and diverse vOTUs (Fig. 5). Taurine had the highest peak area among the organosulphur metabolites detected by LC-MS; this aminosulphonic acid is widely synthesized by marine metazoans and algae, reaching its highest concentrations in coastal environments [77–79]. Taurine degradation was coupled to the inorganic sulphur cycle through the release of sulphite. Similarly, *Rhizobiales* and *Rhodobacterales* were enriched in *L*-cysteine degradation, yielding hydrogen sulphide (H_2_S), which is the dominant gas produced in tidal creek sediments [80].

‘Organosulphur volatilization’ was tied to thioether metabolism. *L*-methionine degradation to methanethiol (MT), ammonium, and 2-oxobutanoate was found in 93% of MAGs. This is consistent with previous findings on salt marsh sediments, where 78.4% of bacteria showed potential for MT production from L-methionine [81]. Interestingly, the predicted metabolite fluxes positioned the vOTU-linked orders *Rhodobacterales*, *Caldilineales*, *Desulfobacterales*, and *Woeseiales* among the dominant MT producers (Fig. 7B). While MT was not detected by GC-MS, we found evidence for its abiotic oxidation product, dimethyldisulphide (DMDS) [82]. MT may also be microbially converted to dimethylsulphide (DMS) or methane by community members not represented in our MAGs [81, 83]. Beyond MT production, *Rhodobacterales MAGs* were also capable of dimethylsulphoniopropionate (DMSP) cleavage and demethylation. DMSP is a central compound in *Spartina* spp., where it accounts for up to 86% of the sulphur content in tissues [84]. DMSP degradation by *Rhodobacterales* produces DMS (detected by GC-MS), contributing together with MT and DMDS to the atmospheric sulphur budget [80]. Viral modulation of these volatile organosulphurs can limit their emission rates in salt marshes while retaining precursors of organic sulphur compounds. These observations in tidal creeks partially overlap with those reported for the *Spartina* root zone, where viruses were found to target microbial groups potentially involved in sulphur oxidation, sulphate reduction, and iron oxidation [28].

### Auxiliary viral genes affect host maintenance and resource scavenging

Virus-encoded auxiliary genes (AVGs) are nonessential genes that increase viral fitness by maintaining or manipulating host physiology and metabolism during infection [23]. AVG functions have been characterized for wetland viruses [27], but their roles in salt marsh sediments remain poorly resolved [28, 30]. Hence, we investigated how viruses can modulate host metabolism through AVGs in our tidal creek sediments.

Gene annotation of the 3537 vOTUs with the *phold* tool yielded 49 719 coding sequences (CDSs). Of these, 59% had unknown function; 22% were assigned to viral structural components; 10% to DNA, RNA, and nucleotide metabolism; 5% to Other (OTH, a category possibly including AVGs); 2% to auxiliary metabolic genes and host takeover (AUX); and 2% to other minor categories (Supplementary Files S7 and S8). From an initial set of 191 gene functions belonging to the AUX and OTH categories, we excluded those linked to transport, secretion systems, translation repressors, resistance proteins, DNA modification (methylation, acetylation, glycosylation), and glycan modification. This curated subset was further investigated by searching MAG-encoded proteins via amino acid sequence similarity, linking the BLASTp matches of AVGs to metabolic reactions in GEMs, ultimately defining a set of 50 AVGs. The identified reactions were characterized in the 311 GEMs by stoichiometry, directionality, and pathway context under the assumption that the viral genes under consideration are host-derived and retain their original biochemical function. As a result, we identified two prevalent strategies of metabolic reprogramming: ‘resource scavenging’, the enhanced acquisition of metabolic intermediates crucial to viral replication, and ‘functional host maintenance’, the support of host energy production and redox metabolism.

**Figure 8:**
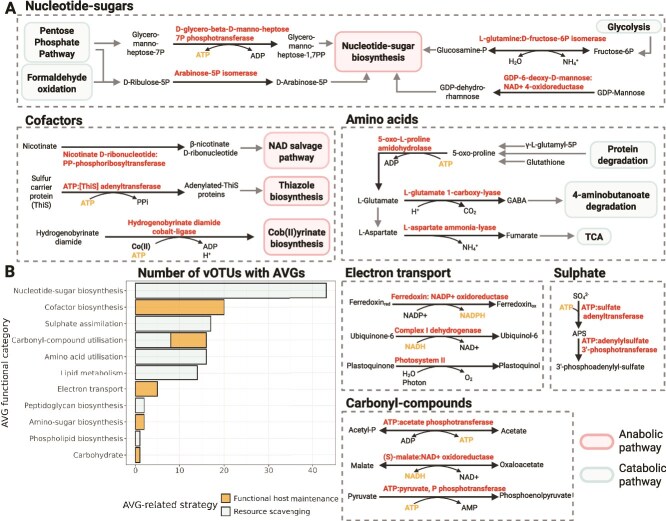
Reaction directionality and metabolic context of auxiliary viral genes matched in GEMs. (A) Reactions in dashed boxes indicate substrates, products, reaction directionality, and pathway connections for auxiliary viral genes (AVGs) matching GEM reactions. Dark arrows indicate the reaction directionality; light arrows indicate metabolic connections inferred from pathway annotation and reaction flux analysis in GEMs. (B) Horizontal bar chart showing the number of vOTUs carrying AVGs reported by AVG functional category; two AVG-related strategies are proposed.

For ‘resource scavenging’, we identified reactions belonging to nucleotide-sugar biosynthesis (34%), amino acid utilization (12%), and sulphate assimilation (4%) (Fig. 8A). ‘Nucleotide–sugar biosynthesis’ genes were the most common AVGs across vOTUs (Fig. 8B). The corresponding proteins catalysed deamination, isomerization, and phosphoryl- or adenylyl-transfer steps in anabolic pathways, using intermediates from glycolysis, pentose phosphate pathway, formaldehyde oxidation, and heterolactic fermentation (Fig. 8A).

These nucleotide-activated sugars serve as precursors of inner core lipopolysaccharides in prokaryotes [85], whereas in viruses they represent the main substrates of phage-encoded glycosyltransferases, which mediate cell-surface remodelling [86], virion decoration [87], and defence evasion by DNA glycosylation [88, 89]. Accordingly, glycosyltransferases were widely detected across vOTUs (122 vOTUs), second only to methyltransferases (512 vOTUs), highlighting how AVGs for nucleotide-sugar biosynthesis support core viral functions.

Six AVGs were involved in ‘amino acid utilization’, with the three most recurrent enzymes being 5-oxo-*L*-proline amidohydrolase, which mediated the conversion of 5-oxo-*L*-proline (produced in glutathione and γ-glutamyl-amino acid metabolism) to *L*-glutamate; and *L*-aspartate ammonia-lyase and *L*-glutamate carboxy-lyase, which mediated amino acid degradation via respiration and fermentation pathways. Substrates and products of these reactions were detected by LC-MS, including 5-oxo-*L*-proline and five γ-glutamyl-amino acids, supporting an efficient scavenging of available amino acids for both biosyntheses and energy production [90, 91].

For ‘sulphate assimilation’, we identified two enzymes participating in sulphate activation: ATP:sulphate adenylyltransferase, which catalyses sulphate attachment to ATP to form adenosine 5′-phosphosulphate, and ATP:adenylsulfate 3′-phosphotransferase, which produces 3′-phosphoadenylsulphate in the following step of the pathway (Fig. 8A). These reactions could be associated with glycan and tRNA modifications [92–94], ATP production [95], or assimilation into amino acids [96]. In this study, galactose-3O-sulphotransferase was the most abundant enzyme in the AUX/OTH category (51 vOTUs); although this annotation may be imprecise given its predominance in eukaryotes, its abundance tentatively indicates glycan modifications as the preferred virus-mediated sulphate utilization mechanism.

For ‘functional host maintenance’, we identified AVGs belonging to the cofactor biosynthesis (14%), carbonyl-compound utilization (10%), and electron transport (8%) categories. ‘Biosynthesis of cofactors’ included seven reactions, particularly involving NAD and cobyrinate. Two NAD biosynthesis reactions converted nicotinamide (detected by LC-MS) to β-nicotinamide-*D*-ribonucleotide and were found in 11 GEMs of *Desulfobacterales* and *Rhodobacterales*. For cobyrinate, one cobalto-chelase catalysed the final step of cob(II)yrinate a,c-diamide biosynthesis, inserting cobalt into the corrin ring (Fig. 8A). Ferredoxin:NADP^+^ oxidoreductase and Complex I dehydrogenase catalysed ‘electron transfer’ to NADP^+^ or NAD^+^, respectively. In addition, one vOTU encoding a photosystem II subunit was matched to a GEM of *Cyanobacteriales*. Altogether, these reactions indicate a capacity of viruses to sustain host redox metabolism through respiratory, electron transport, and photosynthetic functions [97, 98]. In addition, we identified two reactions of ‘carbonyl compounds’ involved in energy production: (S)-malate:NAD^+^ oxidoreductase, which mediated the interconversion of malate and fumarate in TCA variants; and ATP:acetate phosphotransferase, important in pyruvate fermentation to acetate. These generate ATP and NADH, thereby supporting host energy demand during viral infection.

Taken together, the identified AVGs indicate that viruses predominantly modulate the host metabolism by facilitating the biosynthesis and maturation of viral components, exploiting amino acids, nucleotides, carbohydrates, carboxylates, and sulphate as substrates, reflecting the adaptations of their host to the tidal creek habitat [99].

## Conclusion

This study addresses the major challenge of linking microbial diversity and functions to key ecosystem processes in an effort to disentangle the role of microbes in the recovery and preservation of Earth’s ecosystems. To move beyond the diversity assessment, towards a mechanistic interpretation of the viral influence on salt marsh biogeochemistry, we have applied experimental measures, omics approaches, and prediction tools, using genome-scale metabolic modelling and bulk sediment metabolomics to infer substrates and products of microbial metabolism. Our results indicate that viral lysis limits microbial processes such as polysaccharide degradation, organic nitrogen mineralization, and organosulphur mineralization/volatilization. At the same time, auxiliary viral genes for resource scavenging and functional cell maintenance support viral fitness and replication. These effects likely combine with viral shunt and viral shuttle, contributing to nutrient recycling and soil accretion.

All experimental data and statistical–mathematical inferences of this study can be particularly useful to further investigate microbe-mediated SOM transformations, adding stable isotope probing and metatranscriptomics data to fully resolve gene activity, metabolic fluxes, and virus–host linkages, eventually defining integrated community models.

## Supplementary Material

Supplementary_material_ycag184

## Data Availability

The raw sequencing data generated in this study are available on NCBI Sequence Read Archive (SRA) [100] under the Bioproject accession number PRJNA1226540. The metabolomics data have been deposited to MetaboLights [101] repository with the study identifier MTBLS12630. All scripts and genome-scale models are publicly available at https://github.com/r-frizzo/boschettona2023.

## Abbreviations

AMGs, Auxiliary metabolic genes; ANI, Average Nucleotide Identity; APS, Adenosine 5′-phosphosulphate; AUX, Auxiliary metabolic genes and host takeover, a vOTU gene category; AVGs, Auxiliary viral genes; CDS, Coding sequence(s); CHNS, Carbon, Hydrogen, Nitrogen, Sulphur; DMS, Dimethylsulphide; DMDS, Dimethyldisulphide; DMSP, Dimethylsulfoniopropanoate; FBA, Flux Balance Analysis; FC, Fold change; FDR, False Discovery Rate; GABA, Gamma-aminobutyric acid; GC-MS, Gas chromatography–mass spectrometry; GEMs, Genome-scale metabolic models; ICP-MS, Inductively coupled plasma–mass spectrometry; LC-MS, Liquid chromatography–mass spectrometry; MAGs, Metagenome assembled genomes; MT, Methanethiol; NAD, Nicotinamide adenine dinucleotide; OR, Odds ratio; OTH, Other, a category of miscellaneous genes in vOTUs; PA, Peak area(s) of metabolites identified in the sediment; PCoA, Principal Coordinates Analysis; RPM, Reads per million; S, From S1 onwards, S indicates in numerical order a sheet of a supplementary file; SOM, Soil organic matter; TC, Total carbon; TCA, Tricarboxylic acid cycle; TH, Total hydrogen; TN, Total nitrogen; TOC, Total organic carbon; TS, Total sulphur; vOTU, viral Operational Taxonomic Units
