# Supplementary material for: Viral lysis and host reprogramming impact carbohydrate, amino acid, and osmolyte cycling in salt-marsh tidal creek sediments
Source: ISME Commun. 2026 Jul 1;6(1):ycag184. doi: 10.1093/ismeco/ycag184 (PMC13477221; doi:10.1093/ismeco/ycag184)
Supplement: Supplementary file 1 [file supplementary_material_ycag184.zip › Supp. file 1_ycag184.docx]

**Supplementary file 1. Sample preparation, data acquisition and processing**

All statistical analyses, data manipulation, and visualisation steps were performed in R 4.4.2 [1] using the tidyverse [2], data.table [3], magrittr [4], ggtree [5], ape [6], ggstatsplot [7], tidytree [8], MicrobiotaProcess [9], and phyloseq [10] packages. All reagents were purchased from Merck KGaA, Darmstadt, Germany, unless otherwise stated. Multiplot figures were arranged with Biorender ([www.biorender.com](http://www.biorender.com)).

**Sample collection**

Sediment samples (0-2.5 cm depth) were collected on June 26^th^, 2023, at 5:30 am, in low tide conditions, from inner tidal creeks of the Boschettona salt marsh, located in the southern Venice lagoon, Italy (45°15’N; 12°12’E). Six sampling points were selected, each in two replicates and at a minimum distance of 2 m from one another. All sediment samples were collected with long nitrile cuff gloves, using aluminium spoons treated with alcohol and UV-irradiated (sinking the spoon into the sediment to a depth of 2.5 cm and rotating it to collect the sediments). For metagenomics, elemental composition, and metabolomics analyses, aliquots of 40 mL of wet sediments were collected, immediately frozen in dry ice, transported, and stored at -80 °C. For viromics and granulometry analyses, aliquots of 400 mL of wet sediments were collected, transported in a portable cooler at 4 °C, and then stored at -25 °C.

# Physico-chemical sediment characterisation

For CHNS analysis, sediment samples were dried overnight at 100 °C, then ground to eliminate aggregates before analysing total carbon (TC), total organic carbon (TOC), hydrogen (TH), nitrogen (TN), and sulphur (TS). For TC, TH, TN and TS, approximately 15 mg of dry sediments were transferred into a tin capsule and accurately weighed; then, 8 mg of vanadium pentoxide was added, and the capsule was sealed. For TOC, approximately 15 mg of dry sediments were transferred into a silver (SÄNTIS Analytical AG, Teufen, Switzerland) and a tin capsule, accurately weighed, treated with HCl 10% to remove inorganic carbon, and dried at 65 °C for 4 h. CHNS analysis was conducted with Flash Smart instrument (Thermo Scientific, Waltham, US), setting the reference He flux to 140 mL/min, carrier He flux to 100 mL/min, O_2_ flux to 205 mL/min, and a runtime of 400 s.

For granulometry measures, ~30 g of wet sediments were treated with 100 mL of 15% hydrogen peroxide until complete reaction. Samples were then treated with 10 mL of sodium hexametaphosphate 6 g/L for 5 h and dried at 70 °C overnight. Dried samples were ground to remove aggregates and analysed with a laser granulometer (LS 13320 XR, Beckman Coulter, Brea, US) equipped with Universal Liquid Module, setting the obscuration target to 10% and the laser lambda to 750 nm. The resulting data was grouped into Wentworth grain size classes and analysed.

For Inductively Coupled Plasma-Mass Spectrometry (ICP-MS)**,** aliquots of ~10 g of sediment were freeze-dried for 24 h and then 100 mg were accurately weighed in a 15 mL polypropylene tube. Samples were added with 0.5 g of HNO_3_ and then put in a bain-marie (100 °C) under stirring for 2 h. After cooling, each resulting solution was diluted to a total volume of 10 mL with an aqueous solution containing 300 µg/L of ^72^Ge, to obtain a 5% HNO_3_ (m/m) solution. Samples were filtered on polytetrafluoroethylene (PTFE) membranes (Phenomenex, 0.45 µm syringe filters). Two procedural blanks were prepared by adding all reactants except for the samples and, finally, all samples were analysed with an Agilent 7700 ICP-MS (Agilent Technologies International Japan, Ltd., Tokyo, Japan) to determine the total content of As, B, Ba, Br, Ca, Ce, Co, Cr, Cu, Fe, Ga, I, K, La, Li, Mg, Mn, Na, Nd, Ni, P, Pb, Rb, S, Sr, Tb, Ti, V, Zn, and Zr. The ICP-MS system was equipped with an octapole collision cell operating in kinetic energy discrimination mode, used to minimise polyatomic and argon-based interferences. Measurements were performed in [He] collision mode. Operating conditions and data acquisition parameters were adapted from a validated protocol [11]. The instrument was tuned daily with a standard tuning solution containing 1 μg/L of ⁷Li, ⁸⁹Y, ¹⁴⁰Ce, and ²⁰⁵Tl (Agilent Technologies, UK). Multielement standard solutions for calibration were prepared in HNO_3_ 5% by gravimetric serial dilution at twelve different concentrations (min. 1 ng/L – max. 2000 mg/L). All regressions were linear with a determination coefficient (R^2^) larger than 0.9999. Concentrations were reported as mg of element per kg dry weight of sediment.

# Sample preparation and data acquisition for metabolomics and elemental analysis

For Liquid Chromatography–Mass Spectrometry **(**LC-MS), aliquots of 1 g of sediment (12 samples) were centrifuged and, after water removal, re-weighed to determine the exact mass of the solid fraction (~0.3 g). The sediment was extracted with 800 µL of a methanol:ultrapure water (3:1) solution, containing 200 µg/L of phenylalanine-d_5_ as internal standard, and 1.2 mL of chloroform. The extraction mixture was vortexed for 30 s before and after the addition of chloroform and then centrifuged for 5 min at 1000 rpm. The methanol:ultrapure water fraction was filtered on 0.2 µm Regenerate Cellulose membrane filters (Phenomenex, 4 mm syringe filters) before injection. Quality control (QC) samples were prepared as a pool of all sediment samples under analysis and extracted with the same protocol. Processed samples were analysed with an Ultimate 3000 UHPLC chromatograph coupled with a Q-Exactive™ hybrid quadrupole-Orbitrap™ mass spectrometer (Thermo Fisher Scientific, Waltham, Massachusetts, USA). A TSKgel Amide-80, 3 µm, 2.0×150 mm (LabService Analytica, Anzola dell’Emilia, Bologna, Italy) column stabilised at 30 °C was used in HILIC mode. Eluents were acetonitrile and 3 mM ammonium acetate at a flow rate of 0.2 mL/min. The gradient was 95% acetonitrile for 3 min, linearly increasing to 70% ammonium acetate in 24 min, 70% constant ammonium acetate for 3 min, and then 95% acetonitrile in 2 min, equilibration for 8 min. Mass spectrometric conditions were: electrospray ionization (ESI) in both positive (+) and negative (-) mode, data dependent acquisition with resolution 35,000 in full MS and 17,500 in MS/MS, AGC target 1·10^6^ in full MS and 1·10^5^ in MS/MS, max injection time of 100 ms, m/z range 70-1000, isolation window 2.0 m/z, collision gas nitrogen, normalized collision energy 25 eV. Data-dependent settings were: Minimum AGC target 9·10^3^ and dynamic exclusion 20s. The capillary voltage was 4.0 kV (+)/ 2.8 kV (-), capillary temperature 300 °C (+)/280 °C (-), and the auxiliary gas was nitrogen at 20 psi. The calibration was performed with a standard solution (Pierce LTQ Velos ESI positive/negative ion calibration solution, Thermo Fisher Scientific), and the software Xcalibur 3.1 was used for instrument control (Thermo Fisher Scientific, Waltham, Massachusetts, USA). The sample measurement order was randomised to avoid experimental drift.

For Gas Chromatography–Mass Spectrometry (GC-MS), aliquots of 2 g of homogenised sediment samples (12 samples), with the addition of 50 µL of n-decane (1 mg/L) as internal standard, were analysed with a Centri sample preparation platform (Markes International, Bridgend, United Kingdom) coupled with an Agilent 8860 GC system and an Agilent 5977B GC/MSD mass spectrometer (Agilent Technologies GmbH, Waldbronn, Germany). Volatile compounds were extracted and pre-concentrated with the HiSorb sorptive extraction, using a multi-phase standard DVB/CWR/PDMS fiber. Incubation was performed for 60 min at 60 °C, with vial vortexed for 10 s at 400 rpm. Desorption was performed for 10 min at 250 °C. Desorb trap settings were: trap purge time 0.2 min; purge flow 50 mL/min; trap low and high temperature 5 °C and 280 °C, respectively; trap desorption time 3 min with a split flow of 15 mL/min. GC-MS conditions were: Restek Rxi-624SIL MS capillary column (60 m x 250 μm x 1.4 μm, Restek S.R.L., Milano, Italy). Helium was used as a carrier with a flow rate of 1.2 mL/min. The analyses were carried out with a temperature ramp: the starting temperature (40 °C) was held constant for 5 minutes, followed by an increase of 5 °C/min until 320 °C held constant for 5 min with a total run time of 66 min. The mass spectrometer, with an electron impact mode at 70 eV, was set to scan from m/z 40 to 500. Transfer line was set at 260 °C and MS source and quadrupole temperatures were set at 230 and 150 °C, respectively.

**Metabolomics data analysis**

Raw data were converted to ABF format (Reyfics Analysis Base File Converter) and processed with MS-DIAL 4.9 [12]. For LC-MS peak detection, MS and MS2 tolerance were 0.01 Da and 0.025 Da, respectively; minimum peak height 10^4^ (amplitude), mass slice width 0.1 Da, linear weighted moving average as smoothing method, smoothing level 15 scan and minimum peak width 25 scan. For alignment, retention time tolerance was 0.2 min, mass tolerance 0.015 Da, while retention time factor and EI similarity factor were both 0.5. For GC-MS peak detection, minimum peak height was 10^3^, linear weighted moving average as smoothing method, smoothing level 15 scan and minimum peak width 20 scan. For alignment, retention time tolerance was 0.08 min, 70% of EI similarity tolerance. Metabolites were identified at level 2 [13] by comparing experimental mass spectra with MS-DIAL LC-MS reference libraries “ESI(+)-MS/MS from authentic standards (16,481 unique compounds)” and “ESI(-)-MS/MS from authentic standards (9,033 unique compounds)”. Volatile compounds were identified at level 2 with MS-DIAL GC-MS reference libraries “All records with Kovats RI (9,062 unique compounds)” and further confirmed by comparing mass spectra with the NIST reference library (NIST 08, National Institute of Standards and Technology, Gaithersburg, MD, 2008). The feature’s areas were extracted from MS-DIAL, after alignment, identification and normalisation for QCs (using the LOESS normalization) and internal standard (Relative Standard Deviation (RSD%) = 23%), for further analysis. Curation of assignments, molecular classification, and statistical analyses were performed on LC-, GC- and ICP-MS data with MetaboAnalyst 6.0 [14]: metabolites found with LC- and GC-MS were assigned to chemical sub-classes based on HMDB classification [15] using the *Enrichment analysis* module.

# Sample preparation and sequencing for metagenomics and viromics

For metagenomics, 10 sediment samples were thawed and homogenised by manual shaking. DNA was extracted from ~500 mg of wet sediment using the DNeasy PowerSoil kit (Qiagen, Hilden, Germany) following the manufacturer's protocol. DNA concentration and quality were assessed via a NanoDrop ND-1000 UV-Vis spectrophotometer (ThermoFisher, Waltham, USA). DNA samples were submitted to BGI Group (Warsaw, Poland) for library prep and paired-end 150-bp shotgun metagenomic sequencing, according to the DNBSEQ technology. Adapters were removed, and low-quality sequences were filtered with SOAPnuke 2.1.8 [16] (-n 0.01 -l 20 -q 0.3 --adaMR 0.25 --ada_trim --polyX 50 -- minReadLen 150). Reads were co-assembled from 2 × 5 metagenomic datasets using Megahit 1.2.9 [17] (*meta-large* preset, minimum contig length 1000 bp), managed by the SqueezeMeta 1.6.3 pipeline [18]; contig taxonomy was also assigned with SqueezeMeta.

For the enrichment and extraction of virus-like particles, we considered protocols developed by Trubl et al. [19], Hillary et al. [20], Sazinas et al. [21], Adriaenssens et al. [22], Göller et al. [23]. In detail, 250 g of wet sediment per sample (10 samples) were thawed, split in two 500 mL centrifuge tubes, and suspended in 125 mL of a buffer containing 1% potassium citrate, 10% phosphate-buffered saline (PBS), and 150 mM magnesium sulphate (MgSO_4_). Samples were manually shaken vigorously for 30 s and placed in an orbital shaker at 300 rpm for 15 min, 4 °C. Samples were centrifuged for 10 min at 3000 × g, 4 °C, and supernatants were transferred to new centrifuge tubes. These steps were repeated three times, pooling supernatants to a final volume of 375 mL. The pooled supernatants were centrifuged for 10 min at 10000 × g and 4 °C, then filtered with 0.45 µm sterile PES filters. Polyethylene glycol, (PEG - 6,000 MW) and sodium chloride (NaCl) were added to the filtrate (final concentration of 15% w/v and 2% w/v, respectively) to precipitate virus-like particles overnight at 4 °C. Precipitates were recovered by centrifuging tubes for 15 min at 5500 × g and 4 °C, discarding the supernatants. A volume of 1-2 mL of pellet was recovered from each tube, transferred into a 2 mL Eppendorf tube, and centrifuged for 15 min at 12000 × g to obtain a solid pellet, discarding the supernatant. Pellets were resuspended in 750 µL of 1 mg/mL DNase I solution in DNase buffer and incubated for 1 h at 37 °C. The DNase was then inactivated by adding EDTA to a final concentration of 5 mM. DNA was extracted using the ZymoBIOMICS DNA/RNA Miniprep KIT (Zymo Research, Irvine, USA) following the manufacturer’s protocol. The DNA extracted from aliquots of the same sample was pooled, and DNA concentration was assessed via a Qubit 2.0 fluorometer using the Qubit dsDNA HS assay kit (ThermoFisher, Waltham, USA). DNA samples were submitted to BGI Group (Warsaw, Poland) for library prep and paired-end 100-bp shotgun metagenomic sequencing. Libraries were produced successfully with Kapa Biosystems library preparation kit (Roche Molecular Systems, Pleasanton, US) for three samples, and reads were generated according to the DNBSEQ technology. Procedural blanks for metagenomics and viromics were prepared using autoclaved ultrapure water only. Adapters were removed, and low-quality sequences were filtered with SOAPnuke [16] (-n 0.01 -l 20 -q 0.3 --adaMR 0.25 --ada_trim --polyX 50 --minReadLen 100). Reads were individually assembled from 3 viromics datasets using Megahit [17] (*meta-large* preset).

**Analysis of metagenomics data and integration with metabolomics**

Metagenomic reads from each sample were mapped against contigs using coverM 0.7.0 (*contig* module) [24]. Contigs and associated mapping data were then used for binning with SemiBin2 2.1.0 (*Easy co-assembly* mode) [25]. Clustering contig sequences into bins means to reconstruct the genomes originally present in a sample, according to parameters such as composition, coverage, co‑abundance. The binning process can precisely reconstruct entire genomes or broadly assign partial genomes to higher taxonomic categories, depending on the diversity and complexity of all DNA fragments of a sample [26]. Bin quality was assessed with CheckM2 1.0.1 (*predict* module) [27], bins with completeness ≥ 70% and contamination ≤ 10% were retained for downstream analysis [28], dereplicated at 99% ANI using Galah 0.4.0 (*cluster* module *--ani 99 --checkm2-quality-report*) [29] and considered as metagenome-assembled genomes (MAGs). Dereplication reduces the genomes present in the original sample to a non-redundant set, based on high sequence similarity (the threshold of 99% ANI is required for the construction of custom virus-host databases, as predictions depend on strain-level accessory gene content [30,31]. The taxonomic classification of MAGs was performed with GTDB-Tk v2.4.0 (*classify_wf,* database revision 220) [32]. Metagenomic reads were mapped against MAGs to obtain Reads-per-Million (RPM) counts, using coverM (*genome* module) [24]. RPM values were normalised via Hellinger transformation, and reported as relative abundance (%). MAGs were added to our local copy of the iPhoP phage host database (*add_to_db* function, database version *iPHoP_db_Aug23_rw*) to generate a custom host database [31].

Genome-scale metabolic models (GEMs) were built from each MAG using gapseq 1.2 [33]. Reactions and transporters were predicted with the functions *gapseq* *find* and gapseq *find-transport* and used as input for *gapseq draft* to construct the draft models; all steps were run with default parameters. The final step of model construction in gapseq (gap-filling) adds reactions to the model that both enable biomass production and recover likely metabolic capabilities in cases where sequence similarity to known enzyme-coding reference genes approached but did not reach the similarity threshold. Gap-filling of a draft model in gapseq requires a growth medium ideally known to support the organism's growth in vivo [33]. In our work, the medium composition of each draft GEM was predicted with *gapseq medium*, using as input the metabolites identified via LC-MS and GC-MS, with each metabolite assigned a MaxFlux value proportional to its peak area and scaled from 0.1 to 10 mmol g_DW_^-1^h^-1^. This approach allows to integrate environmental specifications into the models, thus reducing the uncertainty of predictions [34,35].

**Pathway enrichment and metabolite utilisation analysis**

Enrichment of metabolic pathways represented in MAGs was assessed at the level of taxonomic orders by *gapseq find*, using a two-sided Fisher's exact test. Only bacterial orders represented by more than 5 MAGs were retained. P-values were corrected for multiple comparisons using the Benjamini-Hochberg false discovery rate (BH-FDR) procedure applied per pathway across orders. Pathways were considered significantly enriched in a bacterial order when BH-adjusted p < 0.05 and odds ratio > 2. Among enriched pathways, those linked to catabolic and energy-utilisation functions were further analysed.

For metabolite utilisation, Flux Balance Analysis (FBA) was performed for each GEM using cobraR v0.1.1 [36,37]**,** solving for biomass growth. Bacterial orders with at least 5 GEMs, and metabolites exchanged in at least 10 GEMs were considered in downstream analyses. We summarised metabolite exchanges by taxonomic order, calculating (i) exchange frequency (proportion of GEMs importing or exporting each metabolite) and (ii) exchange flux (median import/export flux, negative = import, positive = export). Metabolite import or export fluxes aggregated by order were tested against the median value of the whole set (Wilcoxon’s Rank sum test, one-sided, *p* < 0.05 *FDR-adjusted*).

**Identification of viral sequences, taxonomy assignment, gene annotation, and host prediction**

Viromics and metagenomics contigs were pooled into a single file (contig pool). Virsorter2 2.2.4 (*include-groups: "dsDNAphage, ssDNA"*) [38] and geNomad 1.7.3 (*end-to-end* workflow*,* filtering preset: *conservative*) [39] were used to screen viral sequences in the contig pool. The quality of the viral sequences identified was assessed using CheckV v1.0.3 (*end_to_end* workflow) [40]. Summary tables from Virsorter2, geNomad, and CheckV were merged and used for selecting viral sequences as follows: (i) contigs longer than 5 kbp, having at least one viral gene, less than 2 host genes, and a geNomad virus score > 0.8 or Virsorter2 score > 0.8 or CheckV completeness > 70; (ii) contigs shorter than 5 kbp and having CheckV completeness > 70. Passed sequences were classified as viral or proviral based on geNomad and CheckV classification and split into separate files. Viral and proviral sequences were independently dereplicated at 95% ANI over 85% of the aligned fraction, relative to the shorter sequence, using dRep 3.4.2 (*dereplicate* module, -pa 0.9 -sa 0.95 -nc 0.85 -cm "larger") [41,42]. Dereplicated viral and proviral contigs were considered as independent viral operational taxonomic units (vOTUs). Metagenomics reads were mapped against vOTUs to obtain Reads-per-Million counts (RPM), using coverM (*contig* module) [24] and adopting 3 RPM as the minimum threshold value. RPM values were normalised via Hellinger transformation, and reported as relative abundance (%). The taxonomic classification of vOTUs was performed with geNomad and vContact 3 3.0.0b61 [43], prioritising vContact’s taxonomy in case of conflicting results. vOTUs were assigned to gene-sharing clusters applying the function *components* (igraph package [44]) on vContact3 graph output; the gene-sharing network was visualised with r-igraph [44]. Host taxonomy and vOTU-MAG associations were determined with iPhoP (*predict* model) [31] using the custom phage-host database. vOTUs genes were annotated using pharokka 1.7.3 (--meta) [45] and phold 0.2.0 [46] sequentially. vOTUs genes belonging to categories “Auxiliary metabolic genes and host takeover” and “Other” were evaluated as auxiliary viral genes (AVGs).

These AVGs were manually checked, excluding functions linked to virus replication and host interaction (e.g., transport, secretion systems, translation repressors, resistance proteins, DNA/RNA metabolism, and glycan modification), and genes at the immediate edge of a viral scaffold representing possible host contaminations. The curated set of AVGs was searched in MAGs by amino acid sequence similarity with blast+ 2.14.1 (blastp function) [47]. Hits with a percentage of identity ≥ 30% over at least 70% of the query and e-value < 10^-5^ were retained. The genomic coordinates of the resulting blastp hits were intersected with the contig-level CDS positions reported by gapseq, using *bedtools intersect* v2.31.1 [48], identifying which annotated genes harboured the sequences matched by AVGs. This step allowed each AVG to be linked to its corresponding metabolic reaction(s) in GEMs. Only matches having identical gene function were retained.

# References

1. R Core Team. R: A Language and Environment for Statistical Computing [Internet]. Vienna, Austria: R Foundation for Statistical Computing; 2021. https://www.R-project.org/

2. Wickham H, Averick M, Bryan J, Chang W, McGowan LD, François R, et al. Welcome to the Tidyverse. J Open Source Softw. 2019;4:1686. https://doi.org/10.21105/joss.01686

3. Barrett T, Dowle M, Srinivasan A, Gorecki J, Chirico M, Hocking T, et al. data.table: Extension of `data.frame` [Internet]. 2024. https://r-datatable.com

4. Bache SM, Wickham H. magrittr: A Forward-Pipe Operator for R. 2022.

5. Yu G, Smith DK, Zhu H, Guan Y, Lam TT-Y. ggtree: an r package for visualization and annotation of phylogenetic trees with their covariates and other associated data. Methods Ecol Evol. John Wiley & Sons, Ltd; 2017;8:28–36. https://doi.org/10.1111/2041-210X.12628

6. Paradis E, Schliep K. ape 5.0: an environment for modern phylogenetics and evolutionary analyses in R. Bioinformatics. 2019;35:526–8. https://doi.org/10.1093/bioinformatics/bty633

7. Patil I. Visualizations with statistical details: The “ggstatsplot” approach. J Open Source Softw. 2021;6:3167. https://doi.org/10.21105/joss.03167

8. Yu G. Data Integration, Manipulation and Visualization of Phylogenetic Trees. New York: Chapman and Hall/CRC; 2022. https://doi.org/10.1201/9781003279242

9. Xu S, Zhan L, Tang W, Wang Q, Dai Z, Zhou L, et al. *MicrobiotaProcess:* A comprehensive R package for deep mining microbiome. The Innovation. 2023;4:100388. https://doi.org/10.1016/j.xinn.2023.100388

10. McMurdie PJ, Holmes S. phyloseq: An R Package for Reproducible Interactive Analysis and Graphics of Microbiome Census Data. PLOS ONE. Public Library of Science; 2013;8:e61217. https://doi.org/10.1371/journal.pone.0061217

11. Tajoli F, Dengo N, Mognato M, Dolcet P, Lucchini G, Faresin A, et al. Microfluidic Crystallization of Surfactant-Free Doped Zinc Sulfide Nanoparticles for Optical Bioimaging Applications. ACS Appl Mater Interfaces. American Chemical Society; 2020;12:44074–87. https://doi.org/10.1021/acsami.0c13150

12. Tsugawa H, Ikeda K, Takahashi M, Satoh A, Mori Y, Uchino H, et al. A lipidome atlas in MS-DIAL 4. Nat Biotechnol. Nature Publishing Group; 2020;38:1159–63. https://doi.org/10.1038/s41587-020-0531-2

13. Alseekh S, Aharoni A, Brotman Y, Contrepois K, D’Auria J, Ewald J, et al. Mass spectrometry-based metabolomics: a guide for annotation, quantification and best reporting practices. Nat Methods. Nature Publishing Group; 2021;18:747–56. https://doi.org/10.1038/s41592-021-01197-1

14. Pang Z, Lu Y, Zhou G, Hui F, Xu L, Viau C, et al. MetaboAnalyst 6.0: towards a unified platform for metabolomics data processing, analysis and interpretation. Nucleic Acids Res. 2024;52:W398–406. https://doi.org/10.1093/nar/gkae253

15. Wishart DS, Guo A, Oler E, Wang F, Anjum A, Peters H, et al. HMDB 5.0: the Human Metabolome Database for 2022. Nucleic Acids Res. 2022;50:D622–31. https://doi.org/10.1093/nar/gkab1062

16. Chen Y, Chen Y, Shi C, Huang Z, Zhang Y, Li S, et al. SOAPnuke: a MapReduce acceleration-supported software for integrated quality control and preprocessing of high-throughput sequencing data. GigaScience. 2018;7:gix120. https://doi.org/10.1093/gigascience/gix120

17. Li D, Liu C-M, Luo R, Sadakane K, Lam T-W. MEGAHIT: an ultra-fast single-node solution for large and complex metagenomics assembly via succinct de Bruijn graph. Bioinformatics. 2015;31:1674–6. https://doi.org/10.1093/bioinformatics/btv033

18. Tamames J, Puente-Sánchez F. SqueezeMeta, A Highly Portable, Fully Automatic Metagenomic Analysis Pipeline. Front Microbiol [Internet]. 2019 [cited 2024 Jan 17];9. https://www.frontiersin.org/articles/10.3389/fmicb.2018.03349. Accessed 17 Jan 2024

19. Trubl G, Roux S, Solonenko N, Li Y-F, Bolduc B, Rodríguez-Ramos J, et al. Towards optimized viral metagenomes for double-stranded and single-stranded DNA viruses from challenging soils. PeerJ. PeerJ Inc.; 2019;7:e7265. https://doi.org/10.7717/peerj.7265

20. Hillary LS, Adriaenssens EM, Jones DL, McDonald JE. RNA-viromics reveals diverse communities of soil RNA viruses with the potential to affect grassland ecosystems across multiple trophic levels. ISME Commun. Nature Publishing Group; 2022;2:1–10. https://doi.org/10.1038/s43705-022-00110-x

21. Sazinas P, Michniewski S, Rihtman B, Redgwell T, Grigonyte A, Brett A, et al. Metagenomics of the Viral Community in Three Cattle Slurry Samples. Microbiol Resour Announc. American Society for Microbiology; 2019;8:e01442-18. https://doi.org/10.1128/MRA.01442-18

22. Adriaenssens EM, Farkas K, Harrison C, Jones DL, Allison HE, McCarthy AJ. Viromic Analysis of Wastewater Input to a River Catchment Reveals a Diverse Assemblage of RNA Viruses. mSystems. American Society for Microbiology; 2018;3:e00025-18. https://doi.org/10.1128/mSystems.00025-18

23. Göller PC, Haro-Moreno JM, Rodriguez-Valera F, Loessner MJ, Gómez-Sanz E. Uncovering a hidden diversity: optimized protocols for the extraction of dsDNA bacteriophages from soil. Microbiome. 2020;8:17. https://doi.org/10.1186/s40168-020-0795-2

24. Aroney STN, Newell RJP, Nissen J, Camargo AP, Tyson GW, Woodcroft BJ. CoverM: Read coverage calculator for metagenomics [Internet]. Zenodo; 2024 [cited 2024 Aug 21]. https://doi.org/10.5281/ZENODO.10531253

25. Pan S, Zhao X-M, Coelho LP. SemiBin2: self-supervised contrastive learning leads to better MAGs for short- and long-read sequencing. Bioinformatics. 2023;39:i21–9. https://doi.org/10.1093/bioinformatics/btad209

26. Han H, Wang Z, Zhu S. Benchmarking metagenomic binning tools on real datasets across sequencing platforms and binning modes. Nat Commun. Nature Publishing Group; 2025;16:2865. https://doi.org/10.1038/s41467-025-57957-6

27. Chklovski A, Parks DH, Woodcroft BJ, Tyson GW. CheckM2: a rapid, scalable and accurate tool for assessing microbial genome quality using machine learning. Nat Methods. Nature Publishing Group; 2023;20:1203–12. https://doi.org/10.1038/s41592-023-01940-w

28. Eisenhofer R, Odriozola I, Alberdi A. Impact of microbial genome completeness on metagenomic functional inference. ISME Commun. 2023;3:12. https://doi.org/10.1038/s43705-023-00221-z

29. Aroney STN, Camargo AP, Tyson GW, Woodcroft BJ. Galah: More scalable dereplication for metagenome assembled genomes [Internet]. Zenodo; 2024 [cited 2024 Aug 21]. https://doi.org/10.5281/ZENODO.10526086

30. George NA, Hug LA. CRISPR-resolved virus-host interactions in a municipal landfill include non-specific viruses, hyper-targeted viral populations, and interviral conflicts. Sci Rep. Nature Publishing Group; 2023;13:5611. https://doi.org/10.1038/s41598-023-32078-6

31. Roux S, Camargo AP, Coutinho FH, Dabdoub SM, Dutilh BE, Nayfach S, et al. iPHoP: An integrated machine learning framework to maximize host prediction for metagenome-derived viruses of archaea and bacteria. PLOS Biol. Public Library of Science; 2023;21:e3002083. https://doi.org/10.1371/journal.pbio.3002083

32. Chaumeil P-A, Mussig AJ, Hugenholtz P, Parks DH. GTDB-Tk v2: memory friendly classification with the genome taxonomy database. Bioinformatics. 2022;38:5315–6. https://doi.org/10.1093/bioinformatics/btac672

33. Zimmermann J, Kaleta C, Waschina S. gapseq: informed prediction of bacterial metabolic pathways and reconstruction of accurate metabolic models. Genome Biol. 2021;22:81. https://doi.org/10.1186/s13059-021-02295-1

34. Marinos G, Kaleta C, Waschina S. Defining the nutritional input for genome-scale metabolic models: A roadmap. PLOS ONE. Public Library of Science; 2020;15:e0236890. https://doi.org/10.1371/journal.pone.0236890

35. Bernstein DB, Sulheim S, Almaas E, Segrè D. Addressing uncertainty in genome-scale metabolic model reconstruction and analysis. Genome Biol. 2021;22:64. https://doi.org/10.1186/s13059-021-02289-z

36. Waschina S. Waschina/cobrar [Internet]. 2024 [cited 2024 Dec 19]. https://github.com/Waschina/cobrar. Accessed 19 Dec 2024

37. Orth JD, Thiele I, Palsson BØ. What is flux balance analysis? Nat Biotechnol. Nature Publishing Group; 2010;28:245–8. https://doi.org/10.1038/nbt.1614

38. Guo J, Bolduc B, Zayed AA, Varsani A, Dominguez-Huerta G, Delmont TO, et al. VirSorter2: a multi-classifier, expert-guided approach to detect diverse DNA and RNA viruses. Microbiome. 2021;9:37. https://doi.org/10.1186/s40168-020-00990-y

39. Camargo AP, Roux S, Schulz F, Babinski M, Xu Y, Hu B, et al. Identification of mobile genetic elements with geNomad. Nat Biotechnol. Nature Publishing Group; 2024;42:1303–12. https://doi.org/10.1038/s41587-023-01953-y

40. Nayfach S, Camargo AP, Schulz F, Eloe-Fadrosh E, Roux S, Kyrpides NC. CheckV assesses the quality and completeness of metagenome-assembled viral genomes. Nat Biotechnol. Nature Publishing Group; 2021;39:578–85. https://doi.org/10.1038/s41587-020-00774-7

41. Olm MR, Brown CT, Brooks B, Banfield JF. dRep: a tool for fast and accurate genomic comparisons that enables improved genome recovery from metagenomes through de-replication. ISME J. Nature Publishing Group; 2017;11:2864–8. https://doi.org/10.1038/ismej.2017.126

42. Roux S, Adriaenssens EM, Dutilh BE, Koonin EV, Kropinski AM, Krupovic M, et al. Minimum Information about an Uncultivated Virus Genome (MIUViG). Nat Biotechnol. Nature Publishing Group; 2019;37:29–37. https://doi.org/10.1038/nbt.4306

43. Bin Jang H, Bolduc B, Zablocki O, Kuhn JH, Roux S, Adriaenssens EM, et al. Taxonomic assignment of uncultivated prokaryotic virus genomes is enabled by gene-sharing networks. Nat Biotechnol. Nature Publishing Group; 2019;37:632–9. https://doi.org/10.1038/s41587-019-0100-8

44. Csárdi G, Nepusz T, Müller K, Horvát S, Traag V, Zanini F, et al. igraph for R: R interface of the igraph library for graph theory and network analysis [Internet]. Zenodo; 2024 [cited 2024 Dec 17]. https://doi.org/10.5281/zenodo.14347716

45. Bouras G, Nepal R, Houtak G, Psaltis AJ, Wormald P-J, Vreugde S. Pharokka: a fast scalable bacteriophage annotation tool. Bioinforma Oxf Engl. 2023;39:btac776. https://doi.org/10.1093/bioinformatics/btac776

46. George Bouras, Susie Grigson, Vijini Mallawaarachchi, Eric Deveaud. PHOLD, a sensitive annotation tool for bacteriophage genomes and metagenomes using protein structural homology [Internet]. https://github.com/gbouras13/phold

47. Camacho C, Coulouris G, Avagyan V, Ma N, Papadopoulos J, Bealer K, et al. BLAST+: architecture and applications. BMC Bioinformatics. 2009;10:421. https://doi.org/10.1186/1471-2105-10-421

48. bedtools: a powerful toolset for genome arithmetic — bedtools 2.31.0 documentation [Internet]. [cited 2025 Dec 10]. https://bedtools.readthedocs.io/en/latest/. Accessed 10 Dec 2025
