# Supplementary material for: Viral lysis and host reprogramming impact carbohydrate, amino acid, and osmolyte cycling in salt-marsh tidal creek sediments
Source: ISME Commun. 2026 Jul 1;6(1):ycag184. doi: 10.1093/ismeco/ycag184 (PMC13477221; doi:10.1093/ismeco/ycag184)
Supplement: Supplementary file 1 [file supplementary_material_ycag184.zip › Suppl. file 8_ycag184.docx]

***Supp. fig. 1*** Heatmap describing the presence-absence of specific viral genes by functional category for all vOTUs. Top annotations indicate the functional category of viral genes. Taxonomical vOTU classes are reported on the right. The functional categories Structural components, Auxiliary genes and host takeover, and Other are expanded into further heatmaps, each one showing the most abundant genes (above the 80^th^ centile) and their distribution across vOTUs. The three barplots at the bottom show the total counts per gene. On average, each vOTU included 14 CDS, half of which were annotated. Of all vOTUs, 73.85% had at least one gene for virus structural components, 56.6% at least one gene for DNA, RNA and nucleotide metabolism, whereas 40.6% and 22.6% of vOTUs had at least one gene of the categories OTH and AUX, respectively.

**Supp. fig. 2** Gene arrow plots of a selection of vOTUs carrying auxiliary viral genes linked to substrate utilisation. Relevant gene functions are reported with labels, and auxiliary functions under consideration are highlighted in colour
